# Quality in care requires kindness and flexibility – a hermeneutic-phenomenological study of patients’ experiences from pathways including transitions across healthcare settings

**DOI:** 10.1186/s12913-024-10545-8

**Published:** 2024-01-22

**Authors:** Sisse Walløe, Malene Beck, Henrik Hein Lauridsen, Lars Morsø, Charlotte Simonÿ

**Affiliations:** 1 Department of Clinical Research, Research Unit OPEN, University of Southern Denmark, J. B. Winsløws Vej 9 a, 3. Floor, 5000 Odense C, Denmark; 2 Department of Physio- and Occupational Therapy, Research- and Implementation Unit PROgrez, Næstved-Slagelse-Ringsted Hospitals, Region Zealand, Fælledvej 2C, 4200 Slagelse, Denmark; 3 Department of Paediatrics, Zealand University Hospital, Sygehusvej 10, 4000 Roskilde, Denmark; 4 Department of Sports Science and Clinical Biomechanics, University of Southern Denmark, Campusvej 39, 5230 Odense, Denmark; 5 Department of Health, Institute of Regional Health Research, University of Southern Denmark, Campusvej 55, 5230 Odense M, Denmark; 6 Research- and Implementation Unit PROgrez, Næstved-Slagelse-Ringsted Hospitals, Region Zealand, Fælledvej 2C, 4200 Slagelse, Denmark

**Keywords:** Quality, Healthcare Transitions, Patient Experience, Phenomenology of Practice

## Abstract

**Background:**

The number of people living with chronic conditions is increasing worldwide, and with that, the need for multiple long-term complex care across care settings. Undergoing transitions across healthcare settings is both challenging and perilous for patients. Nevertheless, knowledge of what facilitates quality during transitions in healthcare settings from the lifeworld perspective of patients is still lacking. Therefore, we aimed to explore the lived experience in healthcare quality for Danish adult patients during healthcare pathways including transitions across settings.

**Methods:**

Within a hermeneutic-phenomenological approach, interviews were conducted with three women and five men with various diagnoses and care paths between 30 and 75 years of age. Data underwent a three phased thematic analysis leading to three themes.

**Results:**

Patients with various illnesses’ experiences of quality of care is described in the themes *being powerless in the face of illness; burdensome access and navigation;* and *being in need of mercy and striving for kindness*. This highlights that patients’ experiences of quality in healthcare pathways across settings interweaves with an overall understanding of being powerless at the initial encounter. Access and navigation are burdensome, and system inflexibility adds to the burden and enhances powerlessness. However, caring care provided through the kindness of healthcare professionals supports patients in regaining control of their condition.

**Conclusions:**

This hermeneutical-phenomenological study sheds light on the lived experiences of people who are at various stages in their care paths with transitions across healthcare settings. Although our findings are based on the lived experiences of 8 people in a Danish context, in light of the discussion with nursing theory and other research, the results can be reflected in two main aspects: I) kind and merciful professional relationships and II) system flexibility including access and navigation, were essential for their experiences of care quality during healthcare transitions. This is important knowledge when striving to provide patients with a clear voice regarding quality in care pathways stretching across settings.

**Supplementary Information:**

The online version contains supplementary material available at 10.1186/s12913-024-10545-8.

## Background

 The number of people living with chronic conditions is increasing worldwide, and with that, the need for multiple long-term complex care across care settings [1]. This also applies to Denmark, where about one-third of all adults have at least one chronic condition [2] and the prevalence of long-term complex care across departments, sectors, etc., is increasing notably [3]. These transitions across healthcare settings, i.e. movement between hospital, general practitioner, and/or care provided by municipalities, are both challenging to patients [4] and potentially perilous [3, 5, 6]. Literature suggests that regardless of illness type, dimensions related to the structuring of healthcare services and interpersonal factors are essential to patients’ experiences of quality of care in pathways including transitions across healthcare services [7–11]. Nonetheless, in a systematic review of the existing literature [12], the studies primarily focused on the lived experiences of patients afflicted by a single medical condition, typically at a particular care phase, and within a limited age range. Thus, knowledge of what facilitates quality of care during transitions in healthcare settings from the lifeworld perspective of patients is still deficient. This aligns with a recent report from Denmark, which emphasizes patients' demands for well-coordinated care and care pathways, along with the need for their concerns to be taken seriously in order to achieve excellence in healthcare. The report also highlights the necessity for additional research [13].

Navigation, including coordination and sharing of information, seems to be an essential aspect of quality of care in pathways across healthcare settings [7]. Studies also indicate that patients need coordination, information sharing, and continuity to feel safe and cared for in their care pathways [14–17]. However, many still experience fragmented care [1, 3, 4], and there seems to be inequity in care quality [18–22]. Our prior work showed that no relevant and sufficiently comprehensive tool is yet available to assess the general patient-experienced quality in these complex pathways (Walløe S, Roikjær SG, Hansen. SMB, Zangger G, Mortensen SR, Korfitsen CB, Simonÿ C, Lauridsen HH, Morsø L: Content Validity of Patient-Reported Measures Evaluating Experiences of the Quality of Transitions in Healthcare Settings: A Scoping Review, submitted). Consequently, giving patients a genuine voice in quality improvement and targeting improvement initiatives to patients’ needs is difficult. We see a need to gain this valuable insight that can help understand patient-experienced quality in healthcare better, inform quality assessment from the patient perspective, and improve clinical practice [23, 24]. This paper focuses on lived experiences of patients receiving care in pathways including transitions across settings within the Danish healthcare system to provide patients with this genuine voice. Therefore, we aimed to explore the lived experience in healthcare quality for Danish adult patients during healthcare pathways, including transitions across settings.

## Methods

### Design

In our study, we conducted a hermeneutic phenomenological exploration grounded in the philosophies and methodologies of phenomenology, with a specific focus on the lifeworld—the world of lived experience [25].

Our approach was guided by Max Van Manen's thoughts, emphasizing the intricate interplay between the natural and reflective attitudes in exploring healthcare experiences. This deliberate choice aimed to transcend conventional perspectives and challenge taken-for-granted assumptions in our understanding of healthcare services.

Our scientific endeavour sought an intimate connection with the lived experiences behind the immanent practices of the body, social interactions, and environment. Applying a phenomenology of practice methodology, we delved into the complex domain of healthcare quality, aiming to elucidate the nuanced elements shaping the patient's experiential reality.

Further, building upon Van Manen's lifeworld existentials—lived body, lived time, lived space, and lived human relations—we utilised a theoretical framework as a lens to explore the multifaceted aspects contributing to the composition of the patient's lived experience [25]. These existentials, rooted in the lifeworld, became integral tools for our reflective data analysis, guiding the identification and description of interconnected facets shaping specific lived experiences.

In summary, our study, rooted in phenomenology of practice and enriched by hermeneutical dimensions, aimed to extend beyond the superficial and gain a nuanced understanding of the patient experience in healthcare. The lifeworld existentials provided a theoretical foundation, offering valuable insights into the various dimensions of lived experiences within the healthcare context. Through this approach, we aimed to contribute to a profound comprehension of the intricate interplay between individuals and the evolving landscape of healthcare services.

### Setting and participants

To reflect the experiences of patients in healthcare transitions broadly, we purposefully selected patients with illnesses related to obstetrics, oncology, neurology, chronic back pain, and mental illness. In addition, different stages of care pathways were sought, ranging from the emergency department to being in recovery at home. Patients were recruited in person from Slagelse Hospital, Odense University Hospital, and the Spine Centre of Southern Denmark. Participants were screened for eligibility by staff at the recruiting hospitals and names were passed on to SW. SW then selected potential participants as described above. We recruited three women and five men ranging in age from 26 to 76 years (Table 1). It is coincidental that more men than women were recruited. The interviews took place from February to May 2022.

**Table 1:** Participants’ contacts with healthcare services

	Hospital	Region/private	Municipality
Informant 1	Neurology, Cardiology, Emergency care	General Practitioner, out-of-hours medical generalist	Home healthcare, occupational therapy
Informant 2	Ophthalmology, emergency care	General Practitioner, private practice ophthalmology	Home healthcare
Informant 3	Gynaecology, oncology, orthopaedic surgery	General Practitioner	Home healthcare, rehabilitation, occupational therapy
Informant 4	Gynaecology, oncology, rheumatology	General Practitioner	Home healthcare, rehabilitation
Informant 5	Psychiatry	District psychiatry	Job centre, other services
Informant 6	Spine centre, Orthopaedic surgery	General Practitioner	Rehabilitation
Informant 7	Obstetrics, neonatology	General Practitioner, out-of-hours generalist	Early-childhood nurse
Informant 8	Orthopaedic surgery	General Practitioner	Job centre

### Interviews

We conducted semi-structured interviews guided by Kvale and Brinkmann [26] to bring forth patients’ lived experiences of the quality of care while being in transitions between healthcare settings. We developed an interview guide which we tested with the first two participants (see Appendix 1). As the interviews yielded rich, relevant data, we did not adapt the interview guide and decided to include the pilot-interviews in the study. The interviews were started with an introduction of the aim of the study followed by three open questions: *“Please tell me about your care path?”; “Please, try describing the quality of your care path for me?”; “Are there any other experiences you would like to share with me?”* The questions had an open, curious point of inquiry to understand the lived experiences of patients’ care transitions and the quality hereof. Descriptions were subsequently accompanied by honest and probing enquiries regarding the uniqueness of the episodes, actions/reactions, and the daily context. Seven interviews were conducted in person (SW) and one was done by telephone. The interviews were recorded and transcribed verbatim. The telephone interview was not recorded, but the interviewer created notes during and immediately after the interview.

### Data analysis

**Fig. 1:**
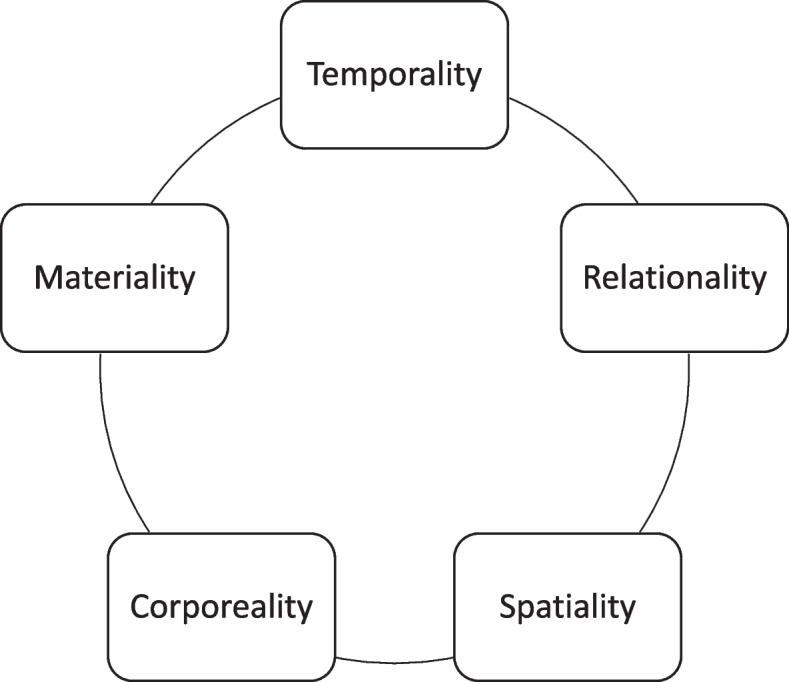
The authors’ depiction of Max van Manen’s existentials [25]

 We conducted a three-phased hermeneutical-phenomenological theme analysis [25] to interpret the interview data (See Table 2 for example). The first author gained a comprehensive understanding of the text through a wholistic reading, familiarizing herself with its overall meaning and grasping phenomena [25] significant to patients’ experiences of being in healthcare transitions. Following this, a selective reading guided by van Manen’s existentials (see Fig. 1) was performed. The existentials supported identification of essential aspects of patient-experienced quality in healthcare transitions and how these phenomena were described [25]. This was done by clustering data that was about lived body to a *corporeality* headline, data material about lived relations was copied to a *relationality* headline, and so forth. In this way, the interview data lent insights into every relevant existential. This constituted the basis of thematic expressions about quality in care. Especially evocative statements were cast to convey the subsequent interpretation [25]. This was done by several readings where the structure of van Manen’s extistentials was slowly erased, and the most significant phenomena stepped forward. A detailed reading was conducted [25], which involved thematic analysis with an in-depth interpretation of the patients’ lived experiences of being in healthcare transitions (See step 3, Table 2). The authors discussed the analysis continuously to achieve agreement on the essence of the participants’ experiences. In the section on findings, we present descriptions of the identified themes. Rhetorical gems from the data material, which we have found especially evocative, are included to give transparency.

**Table 2:** An example of how the 3-step analysis was performed

Step 1	Step 2		Step 3
**Selective thematic basis** Informant 3	**Casting of especially evocative statements**		**Detailed reading with thematic analysis**
For example. I was at the municipal rehabilitation and then I get a call from the hospital. They are saying that I have an appointment with them now. And I say no, I don’t have an appointment. I didn’t get any letter with an appointment. I don’t have e-boks. And then she goes quite. They know I don’t get the digital letters.	Informant 5	INF 5: Well, I go to the district psychiatry every third week, but it is kind of too long for me to be able… it’s as if it sort of crumbles along the way. […] The problem is that it becomes a bit like a garland. Where I kind of go down into depression and start having some psychotic experiences. It is clear that the treatment helps me get back up again, but when the visits are so far apart, I sink pretty low […]. I drop so low that I start having thoughts about suicide and stuff like that.	The participants’ experiences of losing control over their illness were exacerbated by the experience of inflexibility in the provision of healthcare services and how this was perceived as poor quality of care. The participants described feeling powerless towards system rigidness. The expressions are exhaustion, pain, anxiety, and frustration.
It can take all day really. For example, you can leave at half past ten and not be back home until five o’clock. And if you are struggling, or are in pain or something, and have to sit for that many hours, then you can’t do anything the next day. Because all your energy is spent.	Informant 2	So she tells me that she's sent an acute referral to the hospital. And the reply I get is that I can get an appointment in a year. So, I've just been waiting for that. But now, during the last couple of days it's just gotten so bad that I made an emergency call this morning. I am seeing double, my head is spinning, I have pressure to my chest. I Can not wait any longer.	
Of course they are busy people. I know that. I’ve seen how they run around not knowing how to make ends meet. It can be hard for them as well. They are just people like us, aren’t they?	Informant 3	It was Saturday, and all of a sudden they decide to discharge me. My children, they didn’t know when they were going to discharge me.	
It was Saturday, and all of a sudden they decide to discharge me. My children, they didn’t even know they were going to discharge me.	Informant 7	We had been looking forward to going home, and then at 9 the next day, no one shows up. And then we're told that they forgot to sign us up for discharge. So we end up having to wait a day before we can go home. And I just feel like that's not good enough.

Legend Fig. 1: Max van Manen’s existentials [25] as depicted above were used to guide the selective thematic basis of the data. Corporeality is lived body, Materiality is lived things, Temporality is lived time, Relationality is lived human relation, and Spatiality is lived space.

In step 1 clusters of thematic basis were gathered for each participant, supported by the structure of van Manen’s existentials [25]. In step two, especially evocative statements were selected across participants and existentials and cast by thematic expression. The interpretation of the themes was then written in step three. The analysis was an iterative process that moved forwards and backwards between the steps while being discussed in the author group.

## Results

Participants with various diagnoses, illness severity, age, and stages in care gave interviews about their experiences of quality in healthcare pathways including transitions across different settings. Overall, the participants were in a state of powerlessness against illness and in a need for kindness and flexibility when they approached the healthcare system. Their experience of care quality is embedded in this. In the following, this will be unfolded in a description of the three themes identified: *being powerless in the face of illness; burdensome access and navigation;* and *being in need of mercy and striving for kindness.*

### Being powerless in the face of illness

Based on the data, it was identified that participants sought help in the health care system because their ability to function was impaired or threatened, and they lacked the power or resources to help themselves. This was shown when the participants initiated the interview with a disclosure of how or what they were suffering from. Hence, reasons for contacting the healthcare system were based on needing help to manage illness and alleviate symptoms. The participants explained that they had attempted to help themselves or awaited spontaneous alleviation without success. Participants described being out of control with their ill health and needing help from healthcare services to regain the power to manage their everyday lives and cope with illness. The participants expressed this in their narrations of how their illness reduced their ability to take part in meaningful activities such as work, leisure activities, and taking care of depending others. For the most critically ill participants, fear of death was their primary concern.

Participants experienced accentuated powerlessness when their needs were not met in the healthcare system. A man suffering from hallucinations during psychosis explained in these words:

*Formant 5: Well, I go to the district psychiatry every third week, but it is kind of too long for me to be able… it’s as if it sort of crumbles along the way. […] The problem is that it becomes a bit like a garland. Where I kind of go down into depression and start having some psychotic experiences. It is clear that the treatment helps me get back up again, but when the visits are so far apart, I sink pretty low […]. I drop so low that I start having thoughts about suicide and stuff like that.*

The material disclosed how the participants’ experiences of losing control over their illness were exacerbated by the experience of inflexibility in the provision of healthcare services and how this was perceived as poor quality of care. Inflexibility was illustrated by stories of rigid schemas for care that led to expressions of exhaustion, pain, anxiety, and frustration while waiting for care, being transported long distances for care, or being offered a care regime that did not meet the participants’ perceived needs. The participants described feeling powerless towards such rigid schemas and that powerlessness manifested in an experience of being objectified. In objectification, the participants sensed a lack of recognition of their worth as human beings. When the participants’ needs for help were unmet, they were left helpless to suffer alone.

### Burdensome access and navigation

In the participants’ disclosures, experiences of navigating healthcare services were about difficulties in gaining access to care. The participants described this as confusing and stressful. The confusion started when they tried to figure out whom to contact and when to gain access to the help they needed. The more courses of care they had and the more healthcare professionals involved, the more confusing access to care became. Accessing care became even more challenging when the participants’ needs for help appeared suddenly and out of hours, primarily because of a feeling of not fitting in with healthcare service schedules and time slots. Not being able to conform to healthcare time slots made participants circumvent access routes. In this way, they eventually gained access, but the path through the gate had been one of significant energy expenditure and stress. An example of how the participants circumvented access routes was by using emergency contact although they knew they were not in an emergency, or by using their personal network to access care. Below is an example of circumventing access routes:

*Informant 1: Well I know where to go during office hours, but for some reason those painful attacks always hit during the weekend. And the after-hours General Practitioner just isn’t geared for that. They just can’t help and it is so bloody painful. So I call 9-1-1 although I know I’m not really in an emergency. But I need pain relief.*

Gaining access to referrals from the General Practitioner was also experienced as confusing. This scenario was depicted as an experience of being a parcel expedited by one doctor onto the next, which caused a sense of being at risk of stranding in no man’s land. Being in such no-man’s land between referral and accessing care was related to insecurity and unpredictability for participants. This shows how obscurity in referrals made the participants unsure if they would get the help they needed and unable to predict what would come next. Moreover, participants feared being rejected when they tried to get help, and they had concerns about not being taken seriously by the healthcare professionals. The risk of rejection and not being taken seriously was experienced as a lack of acknowledgment that accentuated powerlessness.

After gaining access to care, participants experienced even more challenges in navigating in several cases. A participant with multimorbidity who was admitted to the emergency unit gave an example:

*Formant 1: […] I have my entire calendar booked out with hospital visits, so they will have to discharge me so that I can keep the appointments I’m booked for. I struggle to convey that message to them or to get an answer to how far they have come. […] It feels unsafe. I am the one who has to keep track of all those appointments. And it’s been that way through the whole course of my illness, right from when it started. I am the one who has to be the project manager for my illness.*

The data covers how the participants mainly experienced being left alone with the burden of navigation. Communication flow and coordination between providers were not transparent to the participants. This led to experiences of disrupted and incoherent care in a problematic and inflexible setup that forced participants to be in charge of their care by keeping track of appointments and following up on test results because no one else seemed to be. The perception of trying to navigate a tricky path without a compass or a map was experienced as burdensome and lonely.

In sum, the participants depicted a scenario of overall burdensome navigation in what they perceived as an uncoordinated, inflexible healthcare system, which had the power to abandon them outside the gates – or let them in but not help them. This impacted the perceived care quality negatively.

### Being in need of mercy and striving for kindness

The participants disclosed how they relied heavily on the kindness of the healthcare professionals. In cases where healthcare professionals assumed the responsibility to help the participants compassionately, they felt a burden lifted from their shoulders. The following quote describes the relief the participants felt:

*Formant 7: It was great, such a relief. […] I felt happy after she had been here, even though we agreed I should stop breastfeeding. It was lovely, even though it was actually pretty sad. She was just so smiley and listening and acknowledging. […] She didn’t just weigh him and calculate how much formula he needed. She listened to me and answered the questions I had.*

They experienced being cared for when their needs were met and their suffering was acknowledged. Individual needs being met was illustrated as healthcare professionals adjusting guidelines or bending rules to compensate for the participants’ lack of strength or needs for emotional support from family; that is, according to the participants, acts of kindness. Also, kindness was experienced when healthcare professionals made eye contact, took time to listen, or supported participants’ struggles to cope with suffering. When met with kindness, participants felt less like a burden or an object and more like a respected person. Furthermore, they described regaining the power to help themselves when healthcare professionals facilitated participation and acknowledged their preferences. In this way, the participants were supported back to a position in life where they experienced being more in control, more able to function, and supported in their efforts to live meaningful lives.

The participants’ physical impairments varied, and for those who were most critically ill, caring for their basic functional requirements were imminently significant to their experience of quality. This woman, who had undergone major surgery and was in chemo- and radiation therapy, gave an example of how meaningful the simple act of providing a comfortable chair could be:

*Formant 4: I was SO tired. They planted me in a chair, and I just fell asleep sitting in that chair. […] I have slept in so many waiting rooms simply because I was exhausted. […] So, if I didn’t have to go to all the different hospitals all the time, it would have meant that I could rest, and then I might have had a bit more strength to recover. Maybe I could have better managed it all. If only they had come and said – look, you can have this chair because you can hardly stand on your feet, right?*

This demonstrates that some individuals were barely surviving, struggling to eat, and finding it challenging to take even a few steps. During such times, having a comfortable chair to rest in while waiting at the hospital became the epitome of mercy and excellence in providing care with quality. Participants longed for kindness, through someone to acknowledge and care for their situation, help them compensate, and gain alleviation when they were powerless in the face of illness.

## Discussion

This study highlights that patients with various diagnoses and illness severity, age, and stage in health care pathways experience quality of care across settings to interweave with an overall experience of being powerless at the initial encounter. Of novel interest, quality in care was experienced when patients were met in powerlessness by kindness and flexibility. This will be discussed with a theory of care and other research.

### Kindness facilitates quality in care

We find that patients experience powerlessness before their encounter with healthcare services, and that this powerlessness includes a struggl to cope and find meaning in illness. However, the patients met with kindness and mercy experience regaining control—and in that meaningfulness. The Finish nursing Professor Katie Eriksson denotes suffering in patients as either bearable or unbearable [27]. She stresses that to make suffering bearable, care needs to be caritative with love, mercy, and compassion [28, 29]. The opposite is non-caring care, which accentuates feelings of helplessness, and being trapped in anxiety and shame [29]. In accordance with this, the kindness found in this study can be seen as caritative care [29] that helps reduce powerlessness and fosters regaining control. According to Eriksson caritative care may arise in the encounter between patient and health care professionals [29]. The finding can thus demonstrate that quality in care depends on this interaction.

### The inflexibility of healthcare provision intensifies patients’ powerlessness

In this study, we reveal that patients experience powerlessness and objectification because of rigid, inflexible healthcare services. Hence, patients experience exacerbated powerlessness by being alone with the burden of accessing care and navigating healthcare transitions. In Erikson's view, the powerlessness expressed by the patients may be understood as hopelessness, not only in the face of illness but intensified by the rigidity of healthcare services. This leaves the patients helpless and trapped in shame or exposed to non-caring care, in Eriksson’s words [30]. We interpret that patients are exposed to non-caring care by inflexibility in care pathways across different settings and carrying the burden of challenging access and navigation. This prevents them from regaining control and fosters the experience of poor quality.

### The significance of kindness and flexibility to quality in care

Our findings add new insight into the experienced quality of healthcare pathways across different settings from the patients’ perspective and focuses on care for patients with various illnesses. Several other Danish studies have similar results with transitions described as unsafe and troublesome [31], fragmented [32], or “being lost in a maze” [33], and our findings on patients’ needs for coordination, information sharing, and coherent care pathways are also supported by other research [14–17]. The observation of patients finding navigation of healthcare services burdensome seems to be a global phenomenon [4, 7] resulting in frustration [17] and needs not being met [14, 34]. This indication, that access and navigation are essential to perceived quality of care in healthcare pathways across settings, is further supported by the fact that receiving care across healthcare settings has been found to be a hazard to patient safety [3, 5, 6], especially for the most vulnerable patients [19–21]. This is in line with Beattie et al., who suggest that the Institute of Medicine’s (IOM) conceptualization on quality in healthcare which encompasses safety, timeliness, effectiveness, efficiency, equity, and patient-centeredness [7], could be enhanced by incorporating the concepts of caring and navigation [7]. Our findings, though, add that the system oriented factors safety, timeliness, effectiveness, and efficiency are not sufficient markers of quality in care if not flexible and adaptable to individual patient needs.

The needs for kindness in quality care, which we have found, may be equivalent to the suggestions by Beattie et al. of adding caring to the conceptual definition of quality [7]. This is supported by Baudenstil et al. who found that patients’ preferences for quality indicators across healthcare sectors were mainly focused on human relational themes [35]. Further, Jørgensen et al. found that a trusting relationship with healthcare professionals was meaningful for mental health users to bring coherence to their cross-sectoral care pathways [36]. This finding is similar to ours, but we found the trusting relationship essential for all patients and not just mental health users. Therefor, we infer that patients’ experiences of quality in healthcare transitions are affected by two unique yet interrelated aspects Specifically, one aspect pertaining to the human-relational domain, exemplified by kindness, while the other is referring to the system-related domain, exemplified by flexibility. Given the interdependence we have found between the aspects, we expect a two-factor or bi-factor model to reflect patient-experienced quality in healthcare transitions best. This finding brings valuable information to the development and evaluation of content validity of measures for assessment of patient-experienced quality in healthcare transitions.

### Strengths and limitations

A notable strength is the usage of a hermeneutical-phenomenological approach and letting patients share their perspectives.This enabled us to draw upon patients' lived experiences yielding insights into the dynamics at play during pathways including transitions across healthcare settings among various illnesses, ages, and stages of care. Approaching our data with inspiration from Max van Manen [25] allowed us to focus and study existential aspects of human life with illness, which is needed for healthcare professionals in the future to comprehend what it is like to be an ill person in the world of pathways in the healthcare system. The design also opened new angles. Patient-experienced care quality was linked to powerlessness and the experience of regaining control, by guiding our analysis to see the lived experiences behind the immanent practices of body. Nevertheless, the study results must be interpreted cautiously because they merely reflect experiences within the Danish healthcare system. Moreover, a limitation of this study is, that no participants had other ethnicity than Danish. Further studies, including patients’ experiences from other healthcare systems and with representation of other ethnic groups than Danish, will qualify the knowledge in the field. However, we find the inclusion of participants representing a broad group of people living with different illnesses to be a strength to the transferability of results. Our inclusion strategy may support generalisability and an understanding of what it is like to live transitions between healthcare settings rather than focus on illness-specific themes. Hence, we see our inclusion strategy as a strength, although more men than women were interviewed. We also acknowledge that to generalise and conclude more broadly, our findings from 8 people must be seen in the light of theoretical frameworks and other research.

## Conclusions

This phenomenological-hermeneutic study of 8 patients of different ages living with various diseases who are in healthcare pathways across settings, shows that they struggle with powerlessness in the face of illness in the initial encounter with the health care system. This powerlessness predicts patients' experience of care quality. Patients experience being supported in regaining control during healthcare transitions when met with caring care characterised by both kindness and system flexibility. The two main aspects of patients’ experience of quality of care in healthcare transitions are; I) kind and merciful professional relationships; II) system flexibility including access and navigation. Both aspects should be represented when assessing the patient-experienced quality of healthcare in transitions. Furthermore, we support the idea that IOM’s dimensions of quality may benefit from adding a dimension related to caring care because healthcare professionals play a crucial role in supporting patients to regain control and thus provide quality care.

### Supplementary Information

**Additional file 1. **

## Data Availability

The datasets generated and/or analysed during the current study are not publicly available due to issues regarding anonymity and the protection of study participant privacy. Data sheets containing anonymized quotes and analyses are available from the corresponding author on reasonable request.

## Abbreviation

IOM: Institute of Medicine
