# Supplementary material for: Quality in care requires kindness and flexibility – a hermeneutic-phenomenological study of patients’ experiences from pathways including transitions across healthcare settings
Source: BMC Health Serv Res. 2024 Jan 22;24:117. doi: 10.1186/s12913-024-10545-8 (PMC10801984; doi:10.1186/s12913-024-10545-8)
Supplement: Supplementary file 1 — Additional file 1. [file 12913_2024_10545_MOESM1_ESM.pdf]

English version October 25th 2023

Interview guide for "Quality in Care requires Kindness and Flexibility – a hermeneutic-phenomenological study of patients' experiences from pathways including transitions across settings."

Sisse Walløe, Malene Beck, Charlotte Simonÿ

Introduction

I am interested in what is particularly important to people who receive treatment, care, and/or rehabilitation in care pathways involving primary care medical centres, hospital/s, and municipalities. My focus is on what affects whether patients experience a care pathway as good or poor, and why.

Interview

- Please tell me about your care path.
    - What is your care path about?
    - Whom have you been in contact with in your care pathway?
        - What are their roles?
- Please try describing the quality of your care path for me.
    - What is it that makes you describe your care path like that (good, poor)?
    - Is there a particular incident that has had a significant effect on your experience of the care path?
- Are there any other experiences you would like to share with me?

Suggestions for probing questions

- You described [incident that was good or poor]. Can you please give me a more detailed description of what happened?
- What is it about that incident that makes it particularly important to your experience of your care path?
- Do you have more examples of incidents that made you find the care pathway good/poor?

- When you say, “that’s not good enough”, can you please explain what you mean?
- When you say that was good, can you please explain what that means?
- How did you feel in that situation?
- You are explaining that [X] is crucial to you. Can you put into more words how this matters to you?
- What would it have taken for you to feel that your care pathway was [good/better/…]?
- How could […] have made a difference to you? Why?
